# Targeting PPAR**γ** Signaling Cascade for the Prevention and Treatment of Prostate Cancer

**DOI:** 10.1155/2012/968040

**Published:** 2012-11-14

**Authors:** Sakshi Sikka, Luxi Chen, Gautam Sethi, Alan Prem Kumar

**Affiliations:** ^1^Department of Pharmacology, Yong Loo Lin School of Medicine, National University of Singapore, Singapore 117597; ^2^Cancer Science Institute of Singapore, Yong Loo Lin School of Medicine, National University of Singapore, Singapore 117599; ^3^School of Biomedical Sciences, Faculty of Health Sciences, Curtin University, Perth, WA 6845, Australia; ^4^Department of Biological Sciences, University of North Texas, Denton, TX 76203-5017, USA

## Abstract

The peroxisome proliferator-activated receptor-gamma (PPAR**γ**) is a member of the hormone-activated nuclear receptor superfamily. PPAR**γ** can be activated by a diverse group of agents, such as endogenous polyunsaturated fatty acids, 15-deoxy-Δ^12,14^-prostaglandin J_2_ (15d-PGJ_2_), and thiazolidinedione (TZD) drugs. PPAR**γ** induces antiproliferative, antiangiogenic, and prodifferentiation pathways in several tissue types, thus making it a highly useful target for downregulation of carcinogenesis. These TZD-derived novel therapeutic agents, alone or in combination with other anticancer drugs, have translational relevance in fostering effective strategies for cancer treatment. TZDs have been proven for antitumor activity in a wide variety of experimental cancer models, both *in vitro* and *in vivo*, by affecting the cell cycle, inducing cell differentiation and apoptosis, as well as by inhibiting tumor angiogenesis. Angiogenesis inhibition mechanisms of TZDs include direct inhibition of endothelial cell proliferation and migration, as well as reduction in tumor cell vascular endothelial growth factor production. In prostate cancer, PPAR**γ** ligands such as troglitazone and 15d-PGJ_2_ have also shown to inhibit tumor growth. This paper will focus on current discoveries in PPAR**γ** activation, targeting prostate carcinogenesis as well as the role of PPAR**γ** as a possible anticancer therapeutic option. Here, we review PPAR**γ** as an antitumor agent and summarize the antineoplastic effects of PPAR**γ** agonists in prostate cancer.

## 1. Introduction

Prostate cancer, an androgen-dependent disease, is one of the leading causes of cancers among men worldwide [1]. While the majority of men are diagnosed at early stages of the disease, a subset develop recurrent and eventually metastatic form of the disease [2]. Conventional chemopreventive measures such as surgical resection or radiotherapy are potentially curative for localized disease; however, it has shown to be of limited effectiveness [3]. On the other hand, advanced prostate cancer is associated with a poor prognosis. Tumor growth is originally androgen dependent. Androgens exert their effects through activation of the androgen receptor (AR), a member of the hormone nuclear receptor superfamily. In the mature prostatic gland, the AR regulates the expression of genes involved in cell division and proliferation of the epithelial cells [4]. For more than 60 years ago, androgen deprivation was established as a form of treatment for advanced incurable prostate cancer. Androgen ablation is a hormonal deprivation therapy where the circulating levels of androgen in the body are reduced [3]. The blocking of androgen stimulation often leads to either a partial or full remission; however, subsequent relapse often occurs and the disease reemerges within a few years in a poorly differentiated, androgen-independent form. Furthermore, androgen ablation therapy results in the development of more aggressive forms of prostate cancer which are independent of androgens for growth [3]. The response rates for the treatment are initially high (70–80%); however, almost all patients relapse and develop hormone-refractory prostate cancer (HRPC), resulting in increased morbidity and death [2]. Therefore, there is currently no effective treatment for such androgen-independent forms of prostate cancer. As a result, there is a great interest in identifying more effective treatment options for prostate cancer and alternative therapeutic strategies [3]. Recent studies have shown participation of the nuclear hormone receptor PPAR*γ* in pathophysiology of prostate cancer and its potential in the development of improved anticancer strategies.

Peroxisome proliferator-activated receptor (PPAR) belongs to the nuclear hormone receptor superfamily of transcription factors that includes 48 human transcription factors whose activity is regulated by direct binding of steroid and thyroid hormones, vitamins, lipid metabolites, and xenobiotics [5, 6]. PPARs function as transactivation factors that heterodimerize with retinoid X receptors (RXRs) upon activation and bind to specific response elements (PPREs) in the target DNA of various target genes [7–10]. PPRE consists of direct repeat (DR) hexameric sequences (AGGTCA), separated by one or two nucleotides (DR-1 and DR-2 element) [11]. Distinct areas such as the DNA binding and the ligand-binding transactivation domains have been identified, and these domains influence the transduction of the PPAR-induced response. 

**Figure 1:**
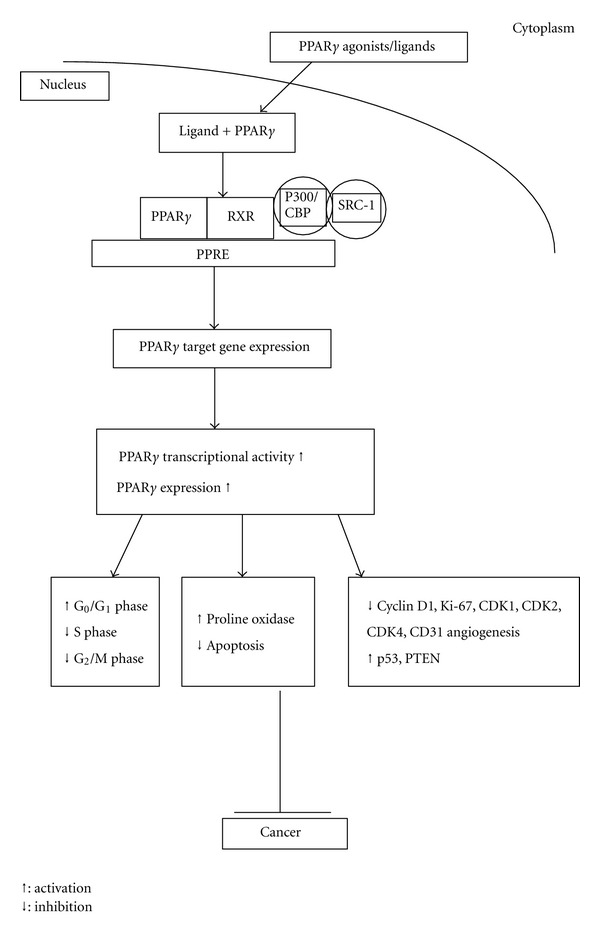
PPAR*γ* activity. Upon ligand binding PPAR*γ*, binds to RXR in the nucleus and associates with coactivators to bind PPRE located on target genes that control various activities.

PPARs have a subfamily of three different isoforms: PPAR*α*, PPAR*β*/*δ*, and PPAR*γ*. In particular, PPAR*γ* plays an important role in the regulation of lipid homeostasis, adipogenesis, insulin resistance, and in the development of various organs. Apart from the established metabolic actions, PPAR*γ* has also been shown to be overexpressed in several types of human cancers, including breast, colon, bladder, and prostate cancer. It was also suggested to induce apoptosis in several malignant cell lineages [12]. In addition, loss-of-function mutations of PPAR*γ* have been found in some human colon and thyroid carcinomas [13]. *In vitro* and *in vivo* studies have demonstrated antiproliferative and proapoptotic actions of PPAR*γ* agonists such as 15d-PGJ_2_ and thiazolidinediones (TZDs) thus suggesting that PPAR*γ* could be a promising therapeutical target for the treatment of cancer [11, 14]. Binding of agonist ligands to PPAR*γ* triggers a conformational change that attracts transcriptional coactivators, including members of the steroid receptor coactivator (SRC) family [5, 15]. Once activated, PPAR*γ* heterodimerizes with retinoid X receptor and signal antiproliferative, antiangiogenic, and prodifferentiation pathways in several tissue types, thus making it a highly useful target for prevention and reduction of carcinogenesis (Figure 1). The synthetic PPAR*γ* ligands, which have been used earlier for the treatment of insulin resistance in type II diabetes mellitus, have been shown to be potential candidates as drugs not only for prevention but also for treatment of prostate cancer [16].

The peroxisome proliferator-activated receptor-gamma has been the focus of intense research during the past decade because ligands for this receptor have emerged as potent insulin sensitizers used in the treatment of type II diabetes. Increased levels of circulating free fatty acids and lipid accumulation in nonadipose tissue have been implicated in the development of insulin resistance. This situation is improved by PPAR*γ* ligands, which promote fatty acid storage in fat depots and regulate the expression of adipocyte-secreted hormones that impact on glucose homeostasis. Insulin resistance is a major defect in type II diabetes mellitus. Synthetic PPAR*γ* agonists, the thiazolidinediones (TZDs), are used in type II diabetes therapy as insulin sensitizers [17] to overcome insulin resistance in target tissues. PPAR*γ* acts as a transcriptional activator of many adipocyte-specific genes involved in lipid synthesis, handling and storage of lipids, growth regulation, insulin signaling, and adipokine production. Thus, PPAR*γ* plays an important role as a major regulator in the differentiation of adipocytes. Thiazolidinediones are characterized by their ability to decrease insulin resistance by specifically targeting at PPAR*γ* to mediate its activity and have been suggested to slow down the progression of insulin resistance [18].

Adipocyte differentiation is a highly regulated process taking place from birth throughout adult. Adipose tissue is composed of adipocytes, which store energy in the form of triglycerides and release it as free fatty acids [19]. Together with muscle, adipose tissue is the major regulator of energy balance of the body. Excessive accumulation of adipose tissue leads to obesity, whereas its absence is associated with lipodystrophic syndromes. PPAR*γ* is highly expressed in the adipose tissue and is required for its development. During adipocyte differentiation, which ensues from PPAR*γ* activation, expression of numerous genes specific for fatty acid metabolism is induced. In fact, functional PPREs have been identified in several genes implicated in adipocyte differentiation, with most of them being involved in lipid storage and control of metabolism. Cell proliferation and differentiation are considered to be mutually exclusive events. However, a close relationship has been established between both cell processes during the adipocyte differentiation program. One of the early events occurring during adipogenesis is the reentry into cell cycle of growth-arrested preadipocytes following hormonal induction. After several rounds of clonal expansion, cells arrest proliferation again and undergo terminal adipocyte differentiation. In the early stage of adipocyte differentiation, an increase in the E2F activity has been observed. E2Fs are transcription factors which regulate the expression of genes involved in DNA synthesis [20]. Consequently, expression of these genes, such as cyclin D1, c-Myc, or cyclin E, is increased in the early stages of adipogenesis [21]. 

It should be noted that neovascularization and adipogenesis are temporally and spatially coupled processes during prenatal life and they continue to reciprocally interact via paracrine signaling systems throughout adult life [22]. Moreover, Biscetti et al. showed that the activation of PPAR*α* and PPAR*γ* leads to endothelial tube formation in an endothelial/interstitial cell coculture assay. This effect was associated with increased production of the angiogenic cytokine vascular endothelial growth factor (VEGF). Neovascularization also occurred *in vivo*, when PPAR*α* and PPAR*γ* agonists were used in the murine corneal angiogenic model. It was concluded that PPAR*α*- and PPAR*γ*-induced angiogenesis is associated with local VEGF production. These findings demonstrate that PPAR*α* and PPAR*γ* activation stimulates neoangiogenesis through a VEGF-dependent mechanism. Neoangiogenesis is a crucial pathological event in type II diabetes. The ability of PPAR*α* and PPAR*γ* agonists to induce neoangiogenesis therefore has important implications for the clinical and therapeutic management of type II diabetes [23]. Thus, it is evident that PPAR*γ* might contribute to protumorigenic process by inducing angiogenesis during the therapy for type II diabetes. However, in this paper we will focus on the anticancer role of PPAR*γ* in aspect of the prevention of prostate cancer. 

PPAR*γ* activation has been implicated to inhibit the proliferation of malignant cells from different lineages such as liposarcoma [24], breast adenocarcinoma [12], prostate carcinoma [25], colorectal carcinoma [26], non-small-cell lung carcinoma [27], pancreatic carcinoma [28], bladder cancer cells [29], and gastric carcinoma cells [30]. Several clinical trials have been initiated that incorporate TZDs for prevention of head and neck cancer or lung cancer. One phase II trial studying the effectiveness of pioglitazone in preventing head and neck cancer in individuals with oral leukoplakia showed that 71% of individuals treated with pioglitazone had complete or partial response, 10% had stable disease, and 19% had progressive disease. Similarly, another clinical trial evaluating the chemopreventive effect of pioglitazone in subjects at risk for lung cancer is currently recruiting participants [31]. Although PPAR*γ* activators have been proven to contribute to anticancer actions during many *in vitro* studies, their advancement into human cancer clinical trials has met with limited success. We will provide an overview of the current findings on PPAR*γ* activation and the targeting in prostate carcinogenesis prevention with the respect of applying PPAR*γ* activators as cancer chemoprevention strategies [32]. There is a demand for safe agents that target high-risk conditions such as preexisting intraepithelial neoplasia, a high-risk prostate cancer precursor. PPAR*γ*-targeted strategies may help to fulfill this demand. As a consequence, PPAR*γ* may be considered an important molecular target for anticancer drug development. 

## 2. PPAR*γ* Ligands (Synthetic and Natural)

There are a variety of endogenous ligands for the PPAR*γ* such as long-chain polyunsaturated fatty acids, arachidonic acid metabolites derived from the cyclooxygenase and lipoxygenase pathways, and fatty acid derived components of oxidized low density lipoproteins (OxLDL) (e.g., 9-hydroxyoctadecadienoic acid and 13-hydroxyoctadecadienoic acid) [33, 34]. The antidiabetic thiazolidinedione (TZD) class of drugs including troglitazone (TGZ), rosiglitazone (BRL49653), pioglitazone, and ciglitazone are synthetic ligands of PPAR*γ*. In addition, nonthiazolidinedione derivatives, such as 2-cyano-3,12-dioxooleana-1,9-dien-28-oic acid (CDDO), CDDO-imidazolide (CDDO-Im), GW-7845, JTT-501, KRP-297, L-764406, MCC-555, GW-0072, and GW-0207 are also synthetic ligands of PPAR*γ* [33]. Most TZDs are selective for PPAR*γ* over the PPAR*α* and PPAR*β*/*δ* subtypes, but there are some exceptions. Thiazolidinedione KRP-297 is a potent PPAR*γ* agonist and a weak PPAR*α* 127 agonist [35]. The PPAR*γ* LBD consists of 13 *α*-helices and a 4-strand *β*-sheet and provides a large binding pocket (1300 Å) that allows access to a number of structurally diverse ligands [36]. The processes of PPAR*γ* activation include interaction with a heat-shock protein as well as cellular signaling that alters the phosphorylation status of PPAR*γ* and the interaction of ligands of both pharmacological and physiological origin. The activation by ligands is the dominant active pathway. After activation, PPAR*γ* forms heterodimer with the RXR and then binds to specific recognition sites, the peroxisome proliferator response elements (PPREs) in the target gene, and regulates transcription of specific genes [34]. Other synthetic compounds that function as ligands include nonsteroidal anti-inflammatory drugs (NSAIDs), such as indomethacin, ibuprofen, flufenamic acid, and fenoprofen. Moreover, there are naturally occurring molecules that function as the endogenous ligands for PPAR*γ*. Among these, the cyclopentenone prostaglandin 15d-PGJ_2_ was found to be the most potent [37]. The natural PPAR*γ* ligand, 15d-PGJ_2_, activates PPAR*γ* at micromolar concentrations in humans *in vivo *[38]. It has been shown that 15d-PGJ_2_ upregulates the expression, transcriptional activity, and DNA-binding activity of PPAR*γ*. 

In addition to synthetic ligands, some natural potent PPAR*γ* agonists from several medicinal plants such as saurufuran A from *Saururus chinensis *(*Saururaceae*), flavonoids such as chrysin and kaempferol, and phenolic compounds from *Glycyrrhiza uralensis *(*Fabaceae*) have also been found [39, 40]. Studies have correlated the consumption of soy-rich diets with a decreased risk of developing hormone-dependent cancers, including prostate cancer [41, 42]. Genistein is a prostate cancer preventive phytochemical found at high levels in soybean and soy foods. To better understand the molecular mechanisms underlying the beneficial effects of genistein on prostate cancer prevention, a DNA microarray approach to examine the effects of genistein at concentrations in the physiologic range on global gene expression patterns in androgen-responsive cancer cells was used. Microarray analyses were performed on androgen-responsive LNCaP human prostate cancer cells exposed to 0, 1, 5, or 25 *μ*M genistein. A concentration-dependent modulation of multiple cellular pathways that are important in prostate carcinogenesis was revealed. The androgen-receptor- (AR-) mediated pathways, in particular, appeared to be modulated by genistein at the lowest concentrations. Furthermore, Lee et al. conducted *in silico* screening for human peroxisome proliferator-activated receptor-gamma (hPPAR*γ*) by performing an automated docking study with 450 flavonoids. 3,6-dihydroxyflavone increased the binding between PPAR*γ* and steroid receptor coactivator-1 (SRC-1) by approximately 5-fold. The 6-hydroxy group of the A-ring of 3,6-dihydroxyflavone participated in hydrogen-bonding interactions with the side chain of Tyr327, His449, and Tyr473. The B-ring formed a hydrophobic interaction with Leu330, Leu333, Val339, Ile341, and Met364. Therefore, 3,6-dihydroxyflavone can be regarded as a potent agonist of hPPAR with cytotoxic effects on human prostate cancer cells [43].

Currently, because of the antitumor and antiangiogenic properties as well as the low toxicity profile, the TZD members have been tested in clinical trials for treatment of human cancers expressing high levels of PPAR*γ* [44]. Evidence indicates that troglitazone and ciglitazone block BH3 domain-mediated interactions between the antiapoptotic Bcl-2 (B-cell leukemia/lymphoma 2) members. Moreover, these TZDs facilitate the degradation of cyclin D1 and caspase-8-related FADD-like IL-l-converting enzyme (FLICE) inhibitory protein through proteasome-mediated proteolysis and downregulate the gene expression of prostate-specific antigen gene expression by inhibiting androgen activation of the androgen response elements in the promoter region [45]. Moreover, TZDs have been proposed in differentiation-mediated therapy of various human carcinomas associated with high levels of PPAR*γ*. Although PPAR*γ* is involved in differentiation and cellular metabolic changes after ligand binding, PPAR*γ*-independent effects have also been described for PPAR*γ* agonists. It has been shown that the naturally occurring PPAR*γ* ligand 15d-PGJ_2_ inhibits the secretion of tumor necrosis factor-*α* (TNF*α*) and interleukin 6 in macrophages stimulated by bacterial lipopolysaccharide and directly blocks activity of the I*κ*B kinase complex in a PPAR*γ*-independent way through inhibition of I*κ*B kinases. Furthermore, it has also been demonstrated that PPAR*γ* ligand troglitazone inhibits cholesterol biosynthesis independently of PPAR*γ* [46]. TZDs have been reported to exert receptor-dependent as well as receptor-independent effects [47]. TZDs can exert receptor-independent effects on several criteria, including that (1) concentrations needed to observe TZD actions were much greater than reported EC_50_ values; (2) the rank order of efficacy for TZDs (troglitazone > pioglitazone > rosiglitazone) to elicit a response was inverse to their known binding affinities (measured EC_50_ values) for PPAR*γ*; (3) effects occurred in the absence of PPAR*γ* expression or of PPAR DNA binding elements (PPRE) in gene promoters. Examples of the receptor-independent effects reported include suppression of inflammatory gene expression, modification of energy and fuel metabolism, suppression of cell proliferation and induction of cytotoxicity, and perturbation of mitochondrial function. Receptor-independent effects of TZDs were also reported in human leukemia (HL) cell lines. In HL-60 cells [48], troglitazone induced cell arrest and subsequent cell death and was associated with downregulation of c-myc, c-myb, and cyclin D2 expression. Since these genes lack a PPRE in their promoter regions, the effect could not directly be PPAR*γ* mediated. Similar findings in a human basophilic leukemia cell line [49] suggested that troglitazone suppressed cell growth independently of PPAR*γ* via a decrease in cyclin E levels and hyperphosphorylation of retinoblastoma tumor suppressor gene product. Moreover, receptor-knockout studies indicate direct evidence that the cytotoxicity induced by TZDs is independent of PPAR*γ*. In PPAR*γ* (−/−) and PPAR*γ* (+/+), mouse embryonic stem cells inhibition of tumor growth by two TZDs, troglitazone and ciglitazone, was independent of PPAR*γ* activity [50]. These compounds blocked the G1/S transition by inhibiting translation initiation as a consequence of partial depletion of intracellular calcium stores and resulting activation of PKR, a kinase that phosphorylates the alpha subunit of eukaryotic initiation factor 2, thus rendering it inactive [51].

Xin et al. first showed that TZDs were potent inhibitors of angiogenesis *in vitro* [52]. The findings indicated that PPAR*γ* is expressed in endothelial cells and its ligands can inhibit endothelial cell proliferation induced by growth factors or cause their apoptosis *in vitro* [52–54]. Furthermore, TZDs inhibit vascular endothelial growth factor (VEGF) and leptin-induced endothelial cell migration [55]. However, clinical studies suggest that TZDs are largely ineffective as monotherapeutic agents in treating prostate carcinoma [56]. Because cancer cells modify several transduction pathways to achieve continuous progression and survival [57], application of multiple drug-strategies appears important to achieve effective treatment. Such a strategy grants synergistic antiproliferative effects and/or permit the use of lower drug doses that might otherwise be less effective when used as monotherapy [58]. 

In addition, activation of PPAR*γ* by pioglitazone caused arrest of exponential growth of fibroblasts and SV40 large T-antigen transformed adipocytes [59]. Examination of the properties of PPAR*γ* in adipocytes suggests that it may be possible to selectively modulate PPAR*γ* activity in an analogous fashion [5]. For example, PPAR*γ* is required for the expression of the adipocyte-specific fatty-acid binding protein aP2 [60]. In mature adipocytes, even in the absence of a pharmaceutical ligand, PPAR*γ* binds to the aP2 promoter along with coactivator proteins [61]. 

It has been reported that binding to helix 12 (H12) of the ligand-binding domain of PPAR*γ* is required for full agonist activity [62]. Previously, the degree of stabilization of the activation function 2 (AF-2) surfaces was thought to correlate with the degree of agonism and transactivation. However, it was observed that the structurally similar PPAR*γ* TZD full agonists rosiglitazone (Avandia) and pioglitazone (Actos) had different clinical adverse events. This indicates that subtle changes in ligand receptor interaction can lead to different pharmacological responses to these agents. As a result, the emphasis has shifted to the development of “selective PPAR*γ* modulators” or SPPARMs. SPPARMs are PPAR*γ* modulators that exhibit potent insulin sensitization activity but are antiadipogenic in animal models of type II diabetes [63–65]. Partial agonists displayed reduced transcriptional activity in reporter assays, and, in animal models of type II diabetes, they demonstrated the SPPARM phenotype. Selective recruitment of transcriptional coactivators has been implicated in partial agonist and SPPARM phenotype. The binding of agonist to the receptor's LBD induces structural changes that facilitate dissociation of repressor molecules (e.g., NCoR and SMRT) and association of activator proteins known to be coactivators to the receptor [66, 67]. These transcriptional coactivators bind to the receptor complex, modify local chromatin structure, and recruit the transcription machinery to target gene promoters [68]. Partial agonists have been shown to have decreased recruitment of CBP (CREB-binding protein) and SRC1 (steroid receptor coactivator-1) coactivators but retain association with PGC1*α* (PPARgamma coactivator-1alpha) [69]. The structure of a partial-agonist-bound PPAR*γ* showed no direct interactions between ligand and H12 [70], supporting the idea that this structural feature is key to maximal transactivation potency of PPAR*γ*. These studies implicated that the degree of H12 stabilization is proportional to the degree of agonism and transcriptional output for full agonists [62].

In prostate cancer, some investigators reported that Telmisartan, an angiotensin II receptor, induced early apoptosis and DNA fragmentation with treatment of 100 *μ*M Telmisartan. These findings indicate that Telmisartan, which is also a selective PPAR*γ* modulator, may mediate potent antiproliferative effects against prostate cancer cells through PPAR*γ* pathway. Thus, Telmisartan could be a potent target for prevention and treatment in prostate cancer [71], while the available data has clearly suggested that PPAR*γ* ligands exhibit potent antiproliferative actions on a wide variety of neoplastic cells. Thus, partial agonists can selectively modulate PPAR*γ* activity by creating interfaces that affect coactivator binding qualitatively as well as quantitatively. In addition, as the corepressors and coactivators interface with overlapping surfaces on the receptor [72], partial agonism may also result from ligand inducing an intermediate conformation that is recognized by both classes of cofactors. Moreover, partial agonists may have full transrepression activity [73], thereby providing a distinct mechanism of selective modulation of PPAR*γ* activity. 

Furthermore, MCC-555, a unique partial agonist of PPAR*γ* as an antidiabetic drug, inhibited the growth of prostate cancer cells both *in* vitro and *in vivo* [74–76]. Several previous reports indicate that, for some types of the PPAR*γ* ligands, their anticancer effects might be independent of PPAR*γ* activation. For example, 15d-PGJ_2_ exerts inhibitory effect against cancer cell proliferation both with and without subjecting to PPAR*γ* activation. In the PPAR*γ*-indpendent mechanism, repression activity of 15d-PGJ_2_ on NF*κ*B-related gene expression was reported, and the action was through covalent modification of critical cysteine residues in I*κ*B kinase and thus preventing the nuclear translocation of NF*κ*B [77]. In addition, some studies revealed that PPAR*γ* classical agonists TZDs (e.g., troglitazone) exhibit anticancer effects via a PPAR*γ*-independent pathway and some non-PPAR*γ* targets such as extracellular signal-regulated kinases, c-Jun N-terminal protein kinase, p38, and Bcl-2 members have been implicated [45, 78].

*In vitro* studies using various solid and hematological tumor cell lines showed that RWJ-241947 had antiproliferative activity against prostate cancer cells, with the strongest effect against the androgen-independent PC-3 prostate cancer cells. RWJ-241947 belongs to the TZD family, and it is established as an antidiabetic drug in animal models of type II diabetes. Like other TZDs, RWJ-241947 binds to PPAR*γ* and exerts transcriptional activities. However, its binding affinity for PPAR*γ* is less than 10% of that of rosiglitazone. Its transcriptional properties are unique because it can function as a full or partial agonist or antagonist, depending on the cell type or DNA-binding site. RWJ-241947 increased expression of cyclin-dependent kinase inhibitor p21^WAF1^, deceased cyclin E, and induced apoptosis in PC-3 cells. On the other hand, RWJ-241947 increased E-cadherin and lowered protein expression of prostate-specific antigen without downregulating the androgen receptor in androgen-dependent LNCaP prostate cancer cells. Reporter gene assays showed that this PPAR*γ* ligand inhibited androgen activation of the androgen receptor response elements of the prostate-specific antigen gene. *In vivo* treatment of male beige/nude/X-linked immunodeficient (BNX) mice with RWJ-241947 profoundly suppressed growth of PC-3 prostate cancer xenografts with prominent apoptosis, as well as fibrosis, including inflammatory and giant cell reaction in the remaining tumor tissue [76]. 

## 3. Effect of PPAR*γ* Ligands on the Proliferation of Prostate Cancer Cells

Inhibition of cell proliferation can occur through regulations in cell cycle and/or apoptosis [3]. c-Myc, a protooncogene product, plays an important role in cell cycle progression, and apoptosis. The c-Myc protein is a basic/helix-loop-helix/leucine zipper transcription factor. It dimerizes with Max and binds specific E-box sequences within DNA and regulates gene transcription. Furthermore, c-Myc regulates expressions of several gene products that are involved in cell proliferation and apoptosis. Expressions of the cyclin dependent kinase (CDK) inhibitors p21 and p27 were downregulated by c-Myc [79, 80]. CDK inhibitors block progression of the cell cycle by inactivating the formation of cyclin/CDK complexes, which are crucial for phosphorylation of retinoblastoma protein when complexed with E2F. Two proteins which promote cell proliferation, cyclin-dependent kinase 4 (CDK4), and the phosphatase CDC25A are also positively regulated by c-Myc [80, 81]. In addition, c-Myc also regulates expressions of proteins that control apoptosis. Expression of the proapoptotic proteins BAD in the rat frontal cortex and BAX in glioblastomas has been shown to be regulated by c-Myc [82]. Akinyeke and Stewart showed that troglitazone not only suppresses prostate cancer cell growth but also decreases c-Myc protein expression [3]. These results suggest that inhibition of c-Myc expression through activation of PPAR*γ* promotes prostate cancer cells to restore characteristics of normal prostate cells phenotype.

PPAR*γ* agonists upregulate CDK inhibitors therefore inducing arrest of the cell cycle. Arrest in G_1_ phase through PPAR*γ* activation has been described in different tumor cell lines [7]. Alterations in p21 and cyclin D1 expression can reduce the phosphorylation of retinoblastoma (Rb) protein, resulting in G_1_ cell cycle arrest. Troglitazone (TGZ) showed a dose- and time-dependent inhibition of the PC-3 cells as examined by MTT assays. In addition, TGZ produced a dose-dependent cell cycle arrest in G_0_/G_1_ of PC-3 cells lines by increasing the distribution of PC-3 cells into the G_0_/G_1_ and sub-G_1_ phase [83]. These discoveries demonstrated that the PPAR*γ*-induced growth inhibition was linked to the G_1_ phase cell cycle arrest through the upexpression of the cyclin-dependent kinase inhibitors p21 and p27 and/or repression of cyclin D1 expression.

Furthermore, to investigate the role of PPAR*γ* in human prostate cancer cells, several ligands of different potency and selectivity were applied to the cell line DU145 and the cell viabilities were assessed after continuous treatment with the drugs. Troglitazone caused a suppression of the growth of these cells, in comparison with vehicle-treated cells. The decrease occurred in a time- and dose-dependent manner, with a 50% inhibition detected at 7 days, at 10 *μ*M concentration [84]. In addition, Yoshimura et al. examined the effects of PPAR*γ* ligands on cell proliferation in prostatic carcinoma (PC) cell lines and investigated the inhibitory effect of troglitazone and 15d-PGJ_2_ on PC-derived cell lines using MTT assay and Hoechst staining. These PPAR-*γ* ligands induced the reduction of cell viability with the half-maximal concentration of growth inhibition of PC cell lines. Furthermore, counting cells at days 1, 2, and 3 clearly showed marked inhibitory effects of PPAR*γ* ligands on cell proliferation [85]. 

Campbell et al. examined the positive association between total fat intake and increased risk for prostate cancer in a case-control study with 175 cases and 233 controls (odds ratio = 1.8, 95% CI = 0.9–3.4) [86, 87]. This study investigated the likelihood that *γ*-tocopherol (GT) could induce growth arrest in PC-3 prostate cancer cells through the regulation of fatty acid metabolism. The findings indicated that GT treatment resulted in upregulation of the PPAR*γ* protein expression at concentrations as low as 5 *μ*M and continued to affect the expression of the PPAR*γ* protein at 40 *μ*M. Growth arrest (40%) and upregulations in PPAR*γ* mRNA and protein expressions were achieved with exposure to GT within 6 h [88]. In addition, expressions of proteins downstream of the PPAR*γ* pathway were also examined. Cyclin D1, cyclin D3, Bcl-2, and NF*κ*B proteins were found to be downregulated following GT treatment. These data demonstrated that the growth arrest mediated by GT follows a PPAR*γ*-dependent mechanism [89]. 

Additionally, GSK-3*β* expression and NF*κ*B activity have important roles in prostate cancer development [90]. To investigate the mechanisms of the PPAR*γ* agonist-induced prostate cancer cell growth inhibition, the authors examined the effect of troglitazone on the expression of PPAR*γ* and GSK-3*β*, activity of NF*κ*B, as well as on the prostate cancer cell growth. Troglitazone induced the expression of PPAR*γ* in the nucleus of PC-3 cells, but not in LNCaP cells. Troglitazone (0–16 *μ*M) inhibited cancer cell growth in both cells accompanied by the induction of cell cycle arrest in G_0_/G_1_ phase and an increased apoptotic cell death in concentration-dependent manner. Troglitazone inhibited the constitutive expression of GSK-3*β* and activation of NF*κ*B. Cotreatment of troglitazone with a GSK-3*β* inhibitor (AR-a014418) or GSK-3*β* siRNA significantly augmented the inhibitory effect of troglitazone on the NF*κ*B activity and on prostate cancer cell growth inhibition and apoptotic cell death. These results suggest that PPAR*γ* agonist, troglitazone, inhibits prostate cancer cell growth through inactivation of NF*κ*B via suppression of GSK-3*β* expression.

## 4. Effect of PPAR-*γ* Ligands on the Apoptosis of Prostate Cancer Cells

It has been shown that PPAR*γ* agonists induce apoptosis. In glioma cells, Zander and coworkers [91] described an upregulation of the proapoptotic proteins BAX and BAD and a functional role of BAX upregulation for the induction of apoptotic cell death. Upregulated expressions of BAD and BAX cause apoptosis by the release of cytochrome C and subsequent activation of several effector caspases [92]. In line with this, PPAR*γ* activation leads to increased caspase 3 activity [7]. Several studies have shown that PPAR*γ* activation leads to inhibition of growth of prostate-cancer cell lines, which is accompanied by morphological changes such as prominent enlarged cytoplasmic vacuoles [7]. TZDs were suggested to exhibit anti-tumor apoptotic effects in human prostate carcinoma (PC) cell lines. Likewise, pharmacological inhibitors of fatty acid synthase (FASN), a metabolic enzyme highly expressed in PC, induce apoptosis in prostate and other cancer cells. A positive correlation between PPAR*γ* and FASN protein in PC cell lines was established, and the synergism between TZDs and FASN blockers in PC cell viability reduction and apoptosis induction was demonstrated. It was concluded that combined treatment of TZDs and FASN has enhanced anti-tumor properties in both androgen-dependent LNCaP and androgen-independent PC-3 and DU-145 cells, when compared with single drug exposure [56].

It has also been shown that the tumor suppressor PTEN (phosphatase and tensin homolog at chromosome ten) possesses PPRE on its promoter and is a PPAR*γ-*targeted gene [93, 94]. PTEN encodes for a phosphatase that dephosphorylates and inactivates kinase PI3K. PI3K through activation of AKT kinase (also known as protein kinase B) inhibits apoptosis. Moreover PPAR*γ* antisense oligonucleotide treatment resulted in significant decrease in caspase 9 activity, further demonstrating the proapoptotic action of PPAR*γ* through a PTEN/caspase mechanism. 

Exposure to the troglitazone has been shown to induce apoptosis in LNCaP, C4-2, and PC-3 prostate cancer cells [3, 95]. The ability of TZDs to promote apoptosis and cell cycle arrest appears to be associated with alterations in protein expressions and activities of antiapoptotic genes. In PC-3 and C4-2 cells, ciglitazone, rosiglitazone, and pioglitazone increased the level of the cyclin-dependent kinase inhibitor p21 [96]. TZDs treatment also stimulated proteasomal degradation of cyclin D1 and *β*-catenin within human prostate cancer cells [96]. In addition, studies have shown decreased phosphorylation and subsequent inactivation of retinoblastoma protein (Rb) in PC-3 cells exposed to ciglitazone. Data from Shiau et al. indicated that troglitazone induces apoptosis in PC-3 cells by reducing the activity of the anti-apoptotic proteins Bcl-2 and Bcl-xL [95].

## 5. Effect of PPAR*γ* Ligands on the Invasion/Metastasis of Prostate Cancer Cells

Metastasis in cancer involves the process of spreading cancerous cells from the original organ or part to another nonadjacent organ or part. PPAR*γ* ligands have been shown to affect endothelial cell proliferation as well as migration and hence regulate angiogenesis [97]. Annicotte et al. have shown that PPAR*γ* activity was repressed by histone deacetylases (HDACs) and enhanced in the presence of HDAC inhibitors. E-cadherin is one of the major factors that inhibit metastasis and invasion of prostate cancer cells through maintenance of the adherens junctions important for epithelial cell-cell adhesion and inhibition of epithelial-to-mesenchymal transition required for cancer progression. Downregulation of E-cadherin expression contributes to oncogenesis [28], and it has been observed to occur in 50% of prostate cancers. Their observations demonstrated that a combination treatment using HDAC inhibitors and PPAR*γ* agonists inhibits invasion of prostate cancer cells *in vivo*, through upregulation of E-cadherin expression [98]. 

Deacetylation of histones has been correlated with a transcriptionally silent state of chromatin. Inhibition of HDAC activity by natural or synthetic compounds results in the reversion of the phenotype of tumoral cells into normal cells or apoptosis in cancer cells [99]. Numerous studies demonstrated that HDAC3 when complexed with PPAR*γ* in the promoters of PPAR*γ*-targeted genes results in gene repression. In PC3 cell line, mRNA and protein expression of the cell cycle inhibitors p19, p21, and p27 were increased in response to pioglitazone, valproic acid, and to a higher extent in the combination treatment. Moreover, cyclin D1 mRNA and protein levels were decreased upon treatment with pioglitazone alone or in combination with valproic acid. 

Moreover, HDAC inhibitors, such as valproic acid or sodium butyrate (NaBu), had a synergistic effect with thiazolidinediones in the activation of PPAR*γ*-targeted genes [100]. This suggests that a cotreatment of HDAC inhibitors and PPAR*γ* agonists potentiates the effects in the arrest of proliferation, increases apoptosis, and decreases the invasion potential of prostate cancer cells. 

## 6. Effect of PPAR*γ* Ligands on the Angiogenesis of Prostate Cancer Cells

Angiogenesis is a physiological process that involves the growth of new blood vessels from preexisting capillaries. Dysregulated angiogenesis can cause many abnormal disorders such as cancer, obesity, arthritis, and blindness. Angiogenesis is regulated by numerous angiogenic factors and mediators. As a major mediator of angiogenesis, vascular endothelial growth factor (VEGF) induces angiogenesis in ischemic or inflamed tissues, wound healing, rheumatoid arthritis, or diabetic retinopathy as well as during carcinogenesis. 

High concentrations of PPAR*γ* are found in tumor endothelium and in healthy skin endothelial cells, and PPAR*γ* activation can induce PPAR*γ* expression in tumor endothelial cells [101]. Earlier study has shown that activation of PPAR*γ* by TZDs inhibits angiogenesis and neovascularization both *in vitro* and *in vivo* and blocks the release of VEGF from smooth muscle cells. Consistent with these data, it has been observed that leptin-induced migration of endothelial cells which is essential for generation of new vessels is inhibited by PPAR*γ* agonists [7]. Interleukin-8 (IL8/CXCL8) is a key effector in prostate cancer progression and contributes to the resistance to standard chemotherapeutic drugs. IL8 belongs to the ELR + CXC chemokine subfamily. It stimulates angiogenesis and has been described as a potent attractant for granulocytic immune cells [2, 102–104]. Like other chemokines that recognize and bind G-protein-coupled receptors, IL8 acts through two receptors, CXC receptor 1 and 2 (CXCR1-2). IL8 has been shown to be involved in prostate cancer progression. Normal prostate epithelial cells and tissues produce low amounts of IL8, whereas prostate cancer cells from primary and metastatic tumors have higher levels of IL8 productions [105–109]. This effect is caused by progressive increases in activation of NF*κ*B transcription factor [110] and correlated to an elevated adherence of the prostate tumor cells to the endothelium [111]. At the cell level, IL8 promotes the transition of prostate cancer to the hormone-refractory prostate cancer (HRPC) state via induction of androgen receptor expression and activation. It stimulates proliferation, invasion, and chemotaxis of HRPC cells through CXCR2 [112]. IL8 has also been involved in PC3 cell tumorigenicity [107], implying that this factor may represent a new molecular target for prostate cancer treatment. 

Also hypoxia-induced angiogenesis can be targeted by PPAR*γ* ligands in cancer therapy, even if the precise mechanisms still remain unclear and require further investigation. As angiogenesis is a crucial aspect for tumor development and metastasis, modulation of angiogenesis by PPAR*γ* ligands would contribute significant clinical benefits in future prostate cancer therapy [97]. 

It is well known that angiogenesis plays an important role in the pathophysiology of ischemic and neoplastic disorders, especially cancer. 15d-PGJ_2_ is involved in regulation of angiogenic mediators including vascular endothelial growth factor and hence participates in the blood vessel formation by means of angiogenesis. 15d-PGJ_2_ inhibits angiogenesis via suppression of proinflammatory enzymes and cytokines, while it also stimulates angiogenesis via induction of heme oxygenase-1, endothelial nitric-oxide synthase, and hypoxia-inducible factor-1*α* [33]. 

## 7. Chemopreventive Effects of PPAR*γ* Ligands in Prostate Cancer Mice Models

Considerable interest has been focusing on TZDs as potential chemopreventive agents in oncology, encouraging observations on the potential anticancer effect of these drugs in several *in vitro *experimental models. Interesting results from animal models studies and in pilot clinical trials have been obtained.

Kubota et al. examined that *in vivo* treatment of PC-3 tumors grown in male BNX triple immunodeficient mice with oral troglitazone (500 mg/kg/day) produced significant inhibition of tumor growth (*P* = 0.01). However, the only objective side effect of troglitazone in mice was the elevation of serum transaminases. Short-term culture of four surgically obtained human prostate cancer tumors with troglitazone (10 microM, 4 days) produced marked and selective necrosis of the cancer cells (about 60%), but not the adjacent normal prostate cells. Taken together, these results suggest that troglitazone may be a useful therapeutic agent for the treatment of prostate cancer, especially in the setting of low disease burden [25]. 

Moreover, troglitazone exhibited a powerful antiproliferative effect on aggressive, androgen-independent PC-3 prostate cancer cells, both *in vitro* and *in vivo* using a murine model. Forty male BNX nu/nu nude mice at 8 weeks of age were used for the experiment. PC-3 cells in 0.1 mL of Matrigel were injected subcutaneously into bilateral sides of each mouse, forming two tumors per mouse. The histological analysis of tumors treated with troglitazone revealed cytological changes of apoptosis including nuclear and cytoplasmic shrinkage and formation of nuclear fragments and apoptotic bodies. In order to determine whether the dramatic effects on PC-3 by troglitazone were mediated through activation of PPAR*γ*, the author also evaluated other PPAR*γ* ligands such as BRL49653, 15dPGJ2, ciglitizone, and indomethacin on prostate cancer cell lines. BRL49653 was more potent than troglitazone in growth inhibition. In addition, 15dPGJ_2_ had a similar potency as troglitazone, and the other two ligands were slightly less potent than troglitazone when treated against PC-3 cells [25]. The TZDs rosiglitazone and troglitazone also reduced growth of LNCaP and PC-3 tumors in nude mouse xenograft models [25, 96, 101]. 

PPAR*γ* has been proven to enhance p53 expression, by binding to the NF*κ*B-responsive element, located in the promoter region of p53 [17, 113]. A recent work by Yu et al. implicated an inhibitory role of PPAR*γ* in hepatocarcinogenesis [114, 115]. In this case, an animal model was used to genetically ablate PPAR*γ* expression on one allele (PPAR*γ* ^+/−^), which decreased PPAR*γ* expression, but was not lethal during embryogenesis as observed in total PPAR*γ* knockout (PPAR*γ* ^−/−^) mice [35, 36]. Using a diethylnitrosamine- (DEN-) induced hepatocarcinoma cell (HCC) model, the authors showed that activation of PPAR*γ* by rosiglitazone blocked tumor development in PPAR*γ* wild-type (PPAR*γ* ^+/+^) littermates, whereas it did not alter tumor formation in PPAR*γ* ^+/−^ mice. To elucidate the underlying mechanism, the authors transduced the human hepatoma cell line Hep3B with a PPAR*γ*-expressing adenovirus. In these transduced cells, PPAR*γ* overexpression induced a G2/M arrest and apoptosis, mediated by extrinsic (Fas and TNF*α*) and intrinsic (caspases 3, 7, 9, and PARP) pathways. Both cell cycle arrest and cell death were enhanced in response to rosiglitazone-mediated PPAR*γ* activation [116–118].

It has been reported that a large proportion of human prostate tumors (40%) carry hemizygous deletions of the PPAR*γ* gene. These findings suggest a functional role of PPAR*γ* as a tumor suppressor gene. Saez et al. concluded that neither hemizygous deletion of PPAR*γ* nor complete ablation of PPAR*α* influenced the development of prostate cancer. In order to elucidate the mechanism of PPAR*γ* signaling in tumor development, strains of mice with defined loss-of-function mutations in the PPAR*γ* gene were generated. Mice devoid of PPAR*γ* gene died *in utero *whereas heterozygotes were viable [119]. 

## 8. Ongoing Clinical Trials with PPAR*γ* Agonists

CS-7017, a Daiichi Sankyo compound, is an oral PPAR*γ* agonist currently undergoing a single arm phase I evaluation [32, 120] for advanced metastatic cancer. A single-arm combination therapy phase I/II clinical trial employing a taxane and CS-7017, for anaplastic thyroid cancer, is also in progress (NCT00603941). There are two recently completed single-arm phase IIa leukoplakia reversal clinical trials, one employing rosiglitazone and the other employing pioglitazone [121]. The pioglitazone clinical trial showed leukoplakia reversal in most patients, and a randomized phase II clinical trial with pioglitazone in leukoplakia patients has been planned [122]. These ongoing or recently completed studies show considerable interest in the clinical study with PPAR*γ* agonists as therapeutic agents.

Thymoquinone, an active ingredient isolated from *Nigella sativa*, was found to induce the activity of PPAR*γ* and PPAR-*β*/*δ* in MCF-7 breast cancer cells [123, 124]. PPAR*γ* has been reported by numerous studies to play a significant role in anticancer mechanisms. By using molecular docking analysis, thymoquinone was shown to interact with seven polar residues and six nonpolar residues in the PPAR*γ* receptor [124]. Thymoquinone-induced apoptosis and decreased surviving levels in MCF-7 cells could be reversed by incubation with GW9662, an irreversible PPAR*γ* inhibitor, suggesting the involvement of PPAR*γ* activity in the anticancer activity of thymoquinone [124].

Recently, a clinical assessment of PPAR*γ* agonists in patients with prostate carcinoma was conducted. In a phase II clinical study, patients with histologically confirmed advanced prostate cancer who had no symptoms of metastatic disease were treated with troglitazone (800 mg per day orally) and showed extended stabilization of prostate-specific antigen (PSA) concentrations, indicating disease stabilization. One patient showed a striking decrease in PSA concentration to almost undetectable amounts [84]. In an index case, a 75-year-old patient with occult recurrent prostate cancer showed a decrease in PSA after oral treatment with troglitazone (600–800 mg per day for 1.5 years) [125]. Segawa and coworkers [126] analyzed prostate tissue from 203 patients and found that PPAR*γ* immunoreactivity was significantly higher in patients with prostate cancer and prostatic intraepithelial neoplasia than in those with benign prostate hyperplasia and in men with healthy prostates. In summary, PPAR*γ* expression is upregulated in prostate cancer and the induction of PPAR*γ* activity provides an additional therapeutic option for treatment of prostate cancer in the near future. 

In conclusion, many earlier studies have shown that TZDs inhibit growth of human prostate cancer cells both *in vitro* and *in vivo* [38]. Two clinical trials treatment with the TZD troglitazone slowed the progression of prostate cancer within patients [84, 125] suggesting that TZDs may serve as effective therapeutic agents for prostate cancer.

## 9. Conclusion and Future Perspectives

Nearly 10 years have passed since the first PPAR subtype was identified. Since then, intense research has led to the development of clinical approaches and synthetic ligands of this nuclear hormone receptor, while some of which are now undergoing clinical trials. Several antineoplastic effects such as induction of apoptosis and differentiation have been described as a result of ligand activation of PPAR*γ* both *in vitro* and *in vivo*.

The mechanisms by which the PPAR*γ* agonists promote apoptosis in cancer cells remain to be fully elucidated. The first few clinical trials to make use of the anti-neoplastic effects mediated by PPAR*γ* have shown conflicting results. On one hand, some studies indicated beneficial effects with PPAR*γ* ligand treatment [42, 43, 80]; however, some other studies could not detect significant anti-neoplastic effects of PPAR*γ* agonists, and the repression effects on tumor growth remain limited [127–129]. Another criticism is that drug concentrations in studies on humans have not been identified. In addition, treatment with a PPAR*γ* agonist might be effective in the prevention of tumor development or might be successful in the treatment of massive tumor growth, if combined with known chemotherapeutics and radiation [7].

Clearly, risk stratification and the targeting of these agents to specific intraepithelial neoplastic conditions will be important in the future testing of these promising chemoprevention drugs. The antiproliferative, prodifferentiation effects of PPAR*γ* activators (TZDs) suggest that these compounds might be useful in slowing the proliferation of undifferentiated tumor cells. Prostate cancers express abundant and higher constitutive levels of PPAR*γ* than do normal prostate cells and are inhibited by ligand activation of PPAR*γ* [130]. These findings have critical implications for the application of PPAR*γ* agonists as potential therapeutic or preventive agents that will spare normal tissue while acting on malignant or premalignant tissue. It is anticipated that PPAR*γ* ligands will provide not only useful mechanistic pathway information but also open a new era of therapeutic options for sufferers of prostate cancer.
